# Supplementary material for: Prognostic Value of EMT Gene Signature in Malignant Mesothelioma
Source: Int J Mol Sci. 2023 Feb 21;24(5):4264. doi: 10.3390/ijms24054264 (PMC10001510; doi:10.3390/ijms24054264)

Supplementary data

Supplementary figure, Figure S1

Strength of prognostic value of EMT gene signature starting from the least significant gene TIMP3 associated with overall survival.

A)Kaplan-Meier survival curves were plotted starting from *TIMP3* with less significant p value (Log-rank p=0.045) and adding one gene at a time up to 9 genes (Log-rank p=4.4e-05). Overall survival (OS) curves show with the HR, a 95% CI, and Log-rank test p values.

B) Strength of prognostic value: starting from *TIMP3* add one gene at a time to calculate the areas under ROC curves up to 9 genes; The AUC of ROC curve is between 0.9-1, suggesting that both sensitivity and specificity are excellent.

A

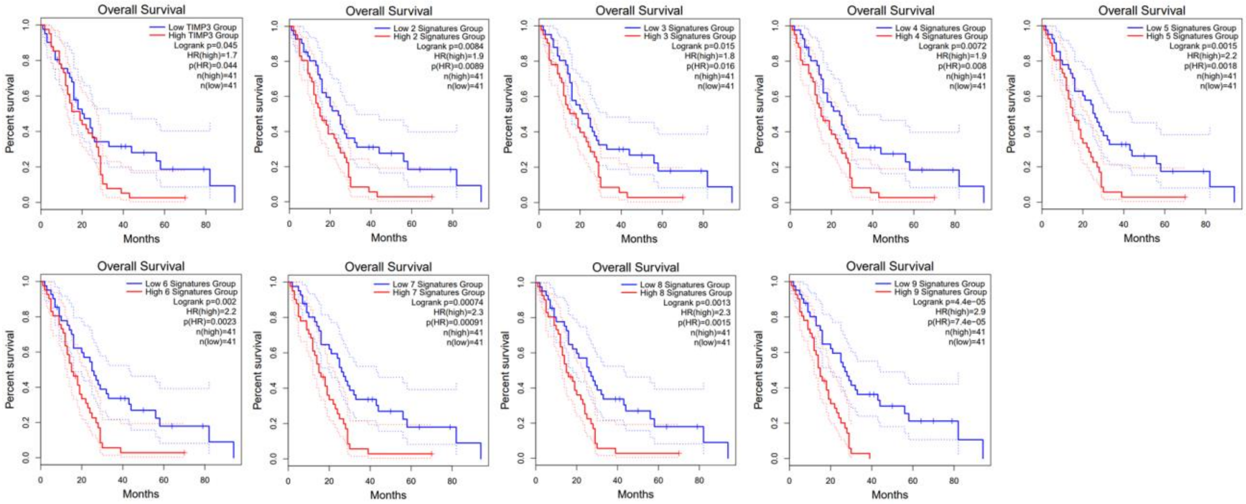

B

Genes	Area under ROC curve	Std. Error	95% confidence interval	P value
1. TIMP3	0.5000	0.04389	0.4140 to 0.5860	>0.9999
2. TIMP3+CALD1	0.7787	0.0352	0.7097 to 0.8477	<0.0001
3. TIMP3+CALD1+ITGAV	0.8103	0.03264	0.7463 to 0.8743	<0.0001
4. TIMP3+CALD1+ITGAV+TPM2	0.8977	0.02291	0.8528 to 0.9426	<0.0001
5. IMP3+CALD1+ITGAV+TPM2+ACTA2	0.9259	0.01855	0.8895 to 0.9622	<0.0001
6. TIMP3+CALD1+ITGAV+TPM2+ACTA2+SERPINH1	0.9790	0.008310	0.9627 to 0.9953	<0.0001
7. TIMP3+CALD1+ITGAV+TPM2+ACTA2+SERPINH1+COL5A2	0.9847	0.006579	0.9718 to 0.9976	<0.0001
8. TIMP3+CALD1+ITGAV+TPM2+ACTA2+SERPINH1+COL5A2+TNFRSF12A	0.9882	0.005292	0.9779 to 0.9986	<0.0001
9. TIMP3+CALD1+ITGAV+TPM2+ACTA2+SERPINH1+COL5A2+TNFRSF12A+SPARC	0.9997	0.0003699	0.9990 to 1.000	<0.0001

Supplementary figure, Figure S2

Strength of prognostic value of EMT gene signature starting from the most significant gene SERPINH1 associated with overall survival.

A) Kaplan-Meier survival curves were plotted starting from *SERPINH1* with most significant p value (Log-rank p=8.5e-06) and adding one gene at a time up to 9 genes (Log-rank p=4.4e-05). Overall survival (OS) curves show with the HR, a 95% CI, and Log-rank test p values.

B)Strength of prognostic value: starting from *SERPINH1* (Log-rank p=8.5e-06) by adding one gene at a time to calculate the areas under ROC curves up to 9 genes; The AUC of ROC curve is between 0.9-1, suggesting that both sensitivity and specificity are excellent.

A

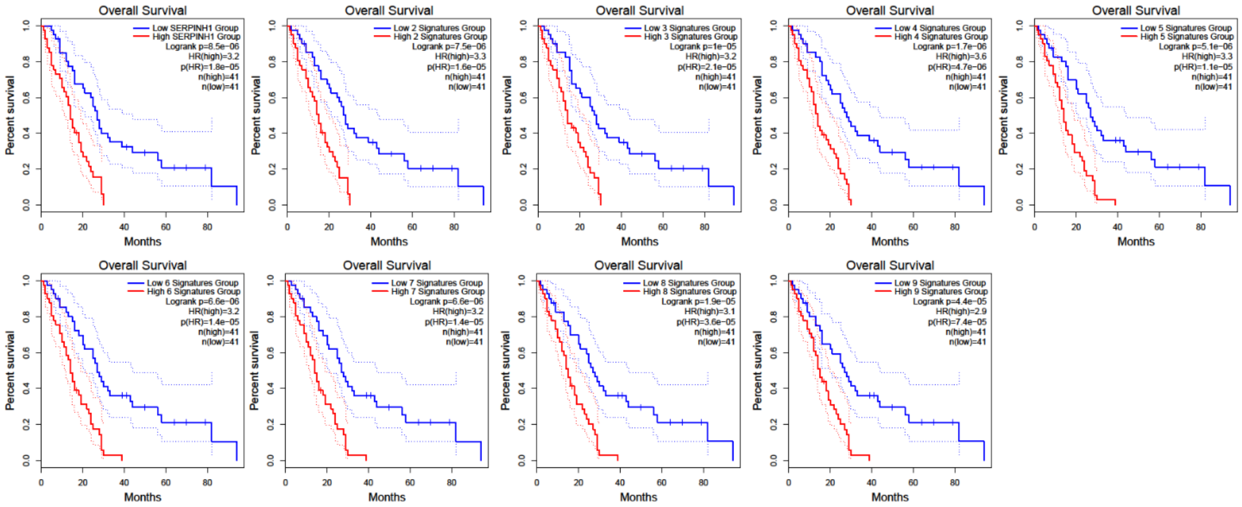

B

Gene	Area under ROC curve	Std. Error	95% CI	p value
1.SERPINH1	0.5	0.04389	0.4140-0.5860	>0.9999
2. SERPINH1+SPARC	0.982	0.007527	0.9673-0.9968	<0.0001
3. SERPINH1+SPARC+TNFRSF12A	0.9877	0.005693	0.9766-0.9989	<0.0001
4. SERPINH1+SPARC+TNFRSF12A+COL5A2	0.9902	0.004954	0.9805-0.9999	<0.0001
5. SERPINH1+SPARC+TNFRSF12A+COL5A2+ACTA2	0.9923	0.004135	0.9842-1.000	<0.0001
6. SERPINH1+SPARC+TNFRSF12A+COL5A2+ACTA2+TPM2	0.9959	0.002657	0.9907-1.000	<0.0001
7. SERPINH1+SPARC+TNFRSF12A+COL5A2+ACTA2+TPM2+ITGAV	0.9964	0.002424	0.9917-1.000	<0.0001
8. SERPINH1+SPARC+TNFRSF12A+COL5A2+ACTA2+TPM2+ITGAV+CALD1	0.998	0.001463	0.9952-1.000	<0.0001
9.SERPINH1+SPARC+TNFRSF12A+COL5A2+ACTA2+TPM2+ITGAV+CALD1+TIMP3	0.9993	0.000701	0.9980-1.000	<0.0001

Supplementary figure, Figure S3

Cancer-associated fibroblast (CAF) cluster may share some EMT gene expression.

Cell type cluster (labelled with Cancer?) was identified as cancer-associated fibroblast (CAF) with high level expression of *S100A4*, *COL5A2*, *FN1*, and *SPARC* but low level expression of *MSLN* and *WT1*.

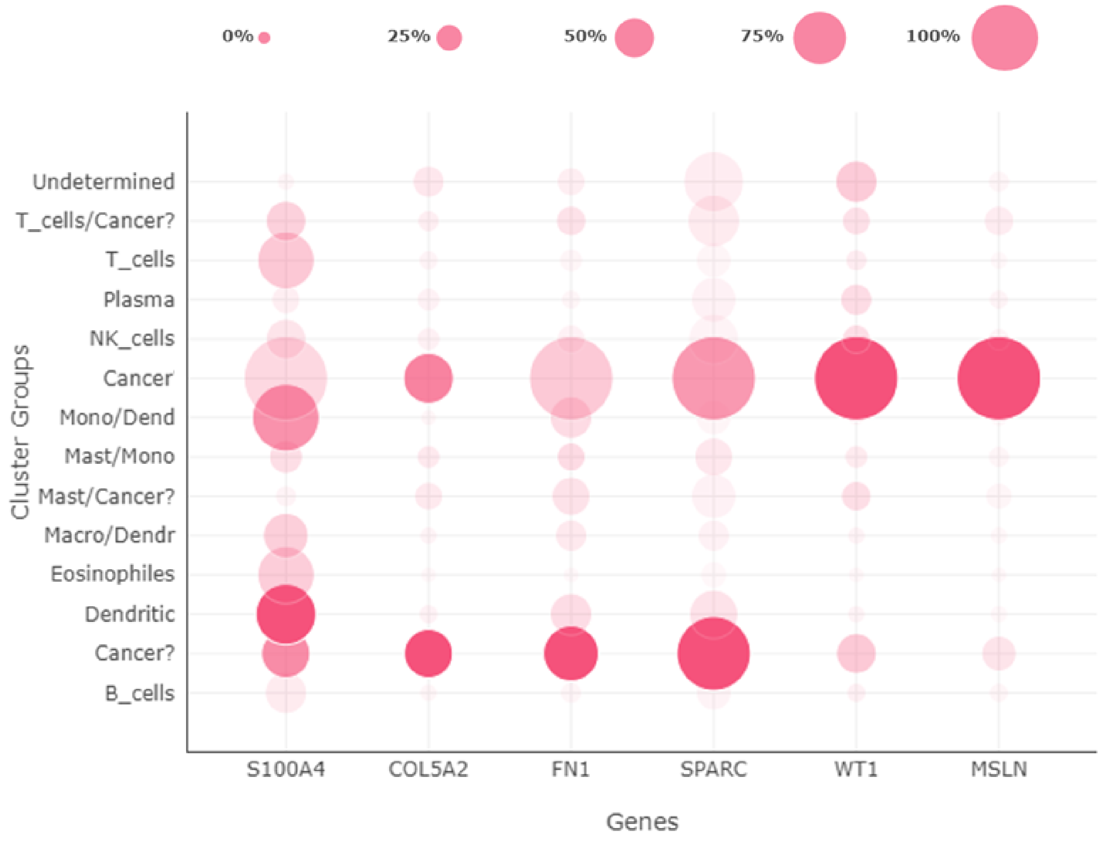

Supplementary figure, Figure S4

Stromal score is an unfavorable factor for prognosis.

A)Stromal scores between epithelioid (n=62) and non-epithelioid (n=25) subtypes in TCGA database.

B)Cancer-associated fibroblast (CAF) infiltration is positively correlated with EMT gene expression in MESO using Timer2.0 and observed a strong correlation in different analytic methods.

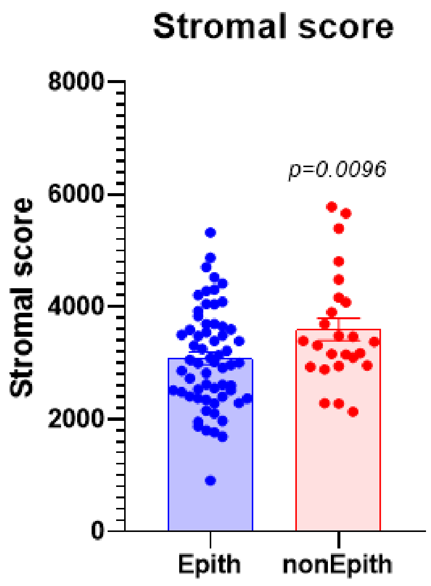

A

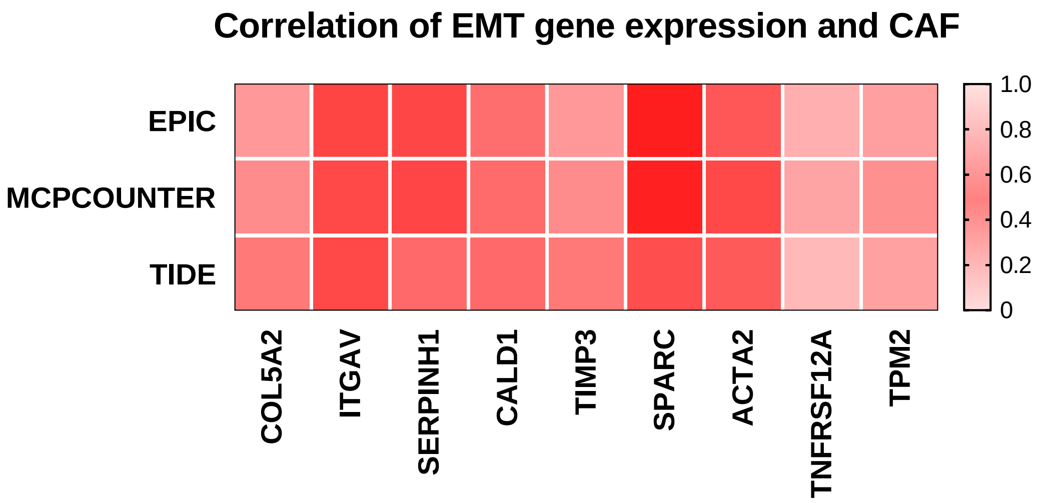

B

Supplementary figure, Figure S5

Proteomic analysis: protein-protein interactions (PPI) of EMT genes determined by NetworkAnalyst.

Generic PPI interaction, IMEx Interactome.The genes of interest (seeds) from previous analysis are mapped to the corresponding molecular interaction database. The procedure typically produces one big subnetwork ("continent") with several smaller ones ("islands"). The size of circles represents the strength of correlation between proteins interaction

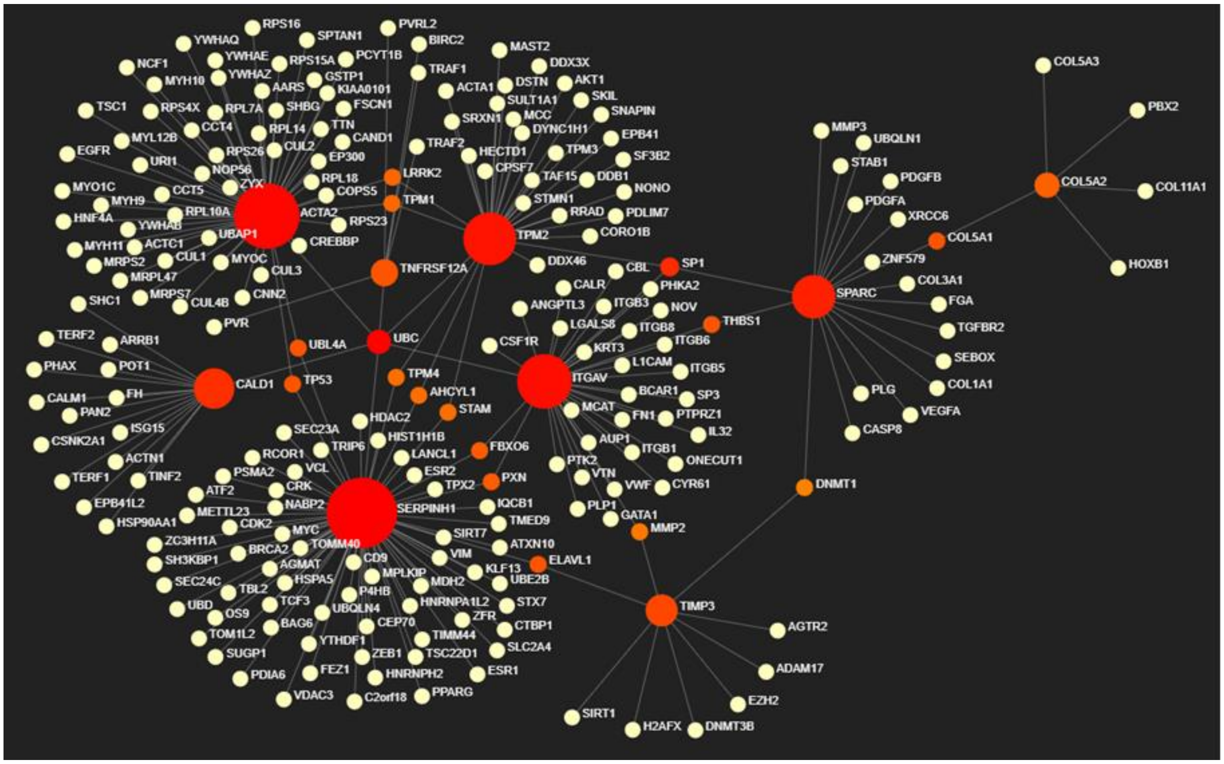

Supplementary figure, Figure S6

Positive correlation between EMT gene expression and immunoinhibitors in MESO.

Immunoinhibitors TGFB1 and TGFBR1 both are positively correlated with EMT genes expression determined by distribution of gene expression across immune cells database (TISIDB), which is an integrated repository portal for tumor-immune system interactions to analyze pan-cancer types from multiple heterogeneous data sets (http://cis.hku.hk/TISIDB/).

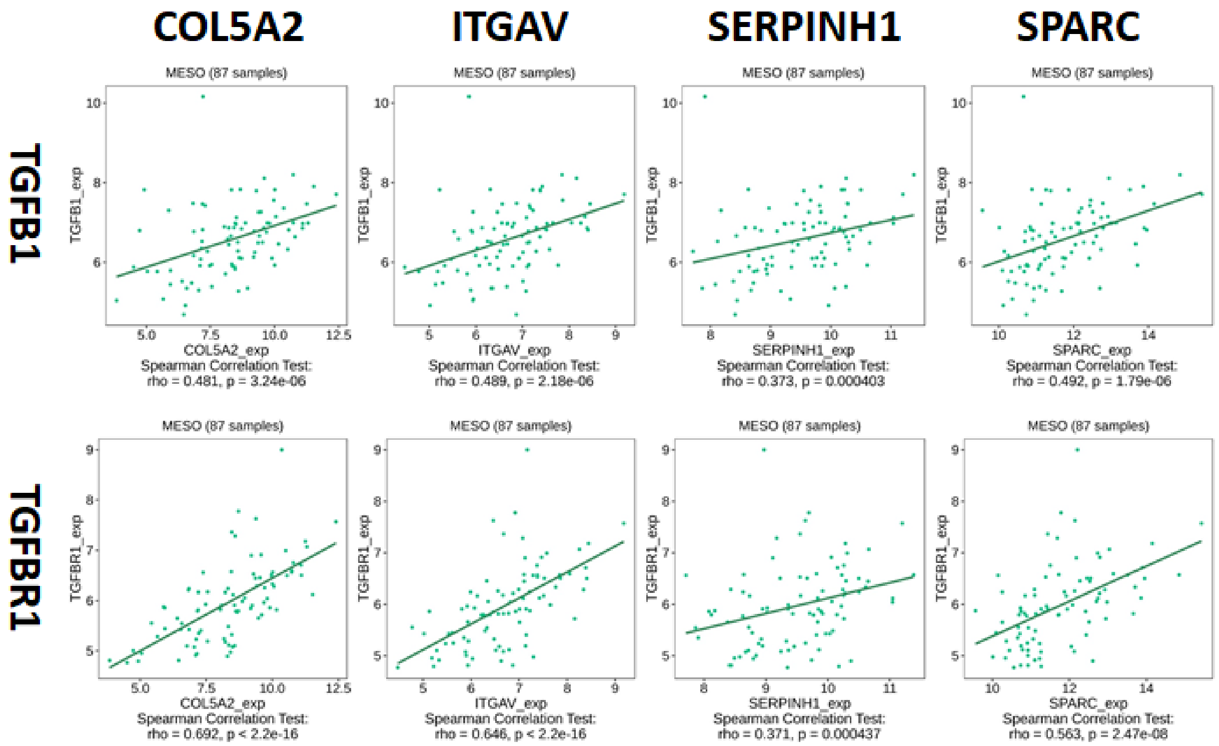

Supplementary figure, Figure S7

Genomic analysis of 9 EMT genes and CDKN2A/B tumor suppressor genes in MESO. Only a few mutations were occurred, including *COL5A2* with missense mutation and *ACTA2* with deep deletion. Frequent deep deletion occurred in CDKN2A/B genes (cbiolportal.org/oncoprinter).

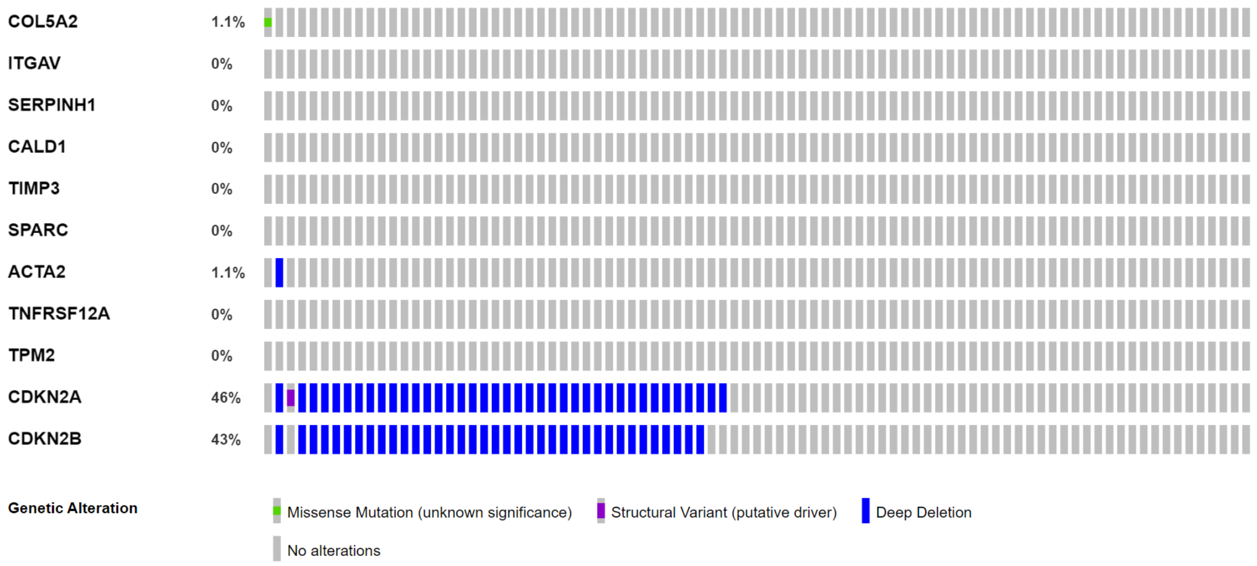

Supplementary figure, Figure S8

Representative genomic and epigenomic alterations, and copy number variation (CNV) of epigenomic CDKN2A/B and EMT genes in MESO.

A) Gene mutations of BAP1, NF2 list in genomic alterations; **B**) DNA methylation of BAP1, NF2 list in genomic alterations. **C**) Gene mutations of EZH2, DNMT1 list in epigenetic genes; **D**) DNA methylation of EZH2, DNMT1 list in epigenetic genes. **E**) Gene mutations of EMT genes; **F**) DNA methylation of EMT genes. **G**) Correlation of D&F DNA methylation. Data were generated from TCGA database from cbiolportal.org platform.

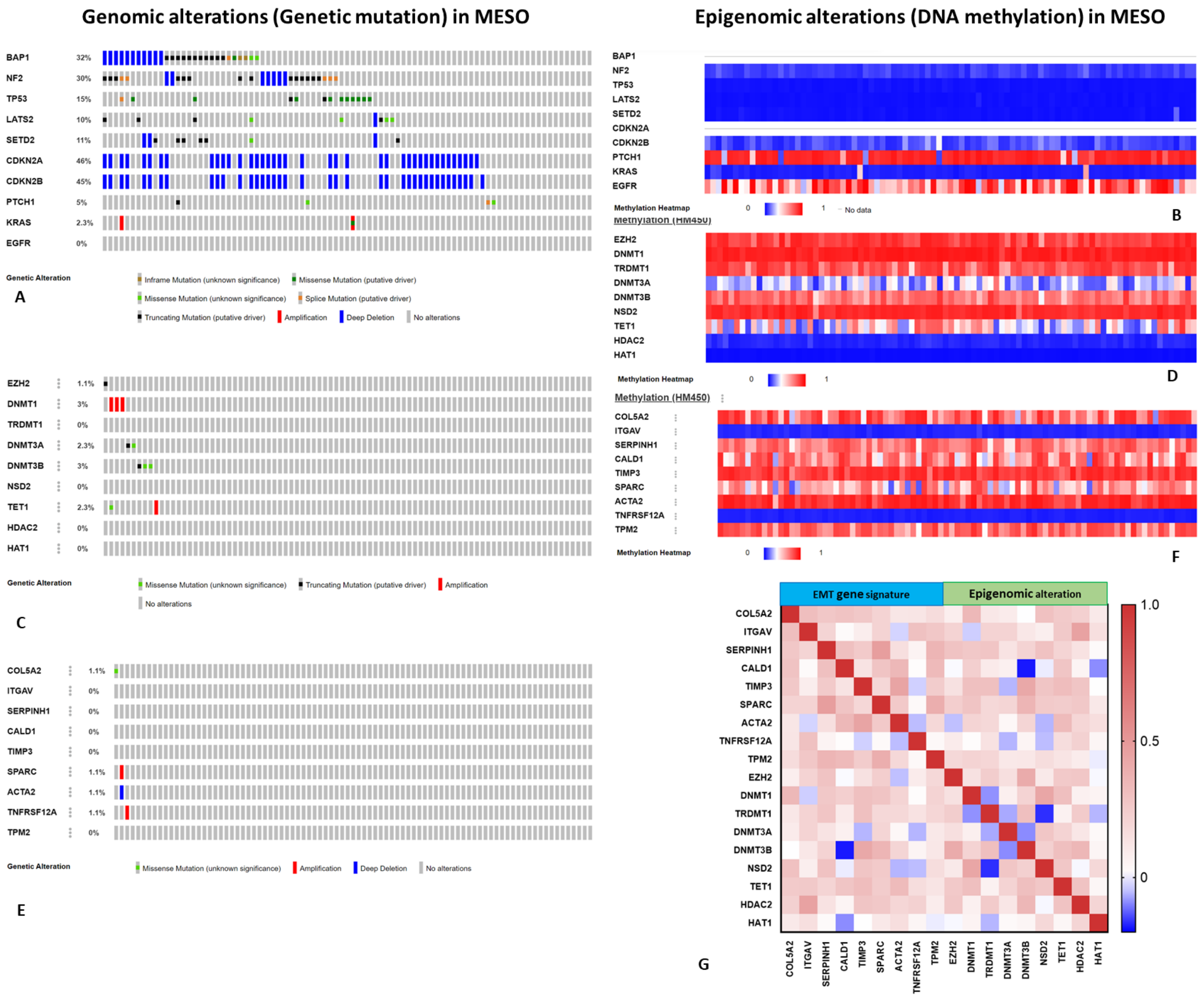

Supplementary figure, Figure S9

The expression of EMT genes is associated with a lack of interferon expression as well as CDKN2A and CDKN2B expression.

A) Heatmap of gene expression in TCGA MESO patients, besides SPARC gene with super high expression, other EMT genes have significantly higher expression levels;

B) Quantification of gene expression in TCGA MESO patients. *** $p < 0.001$

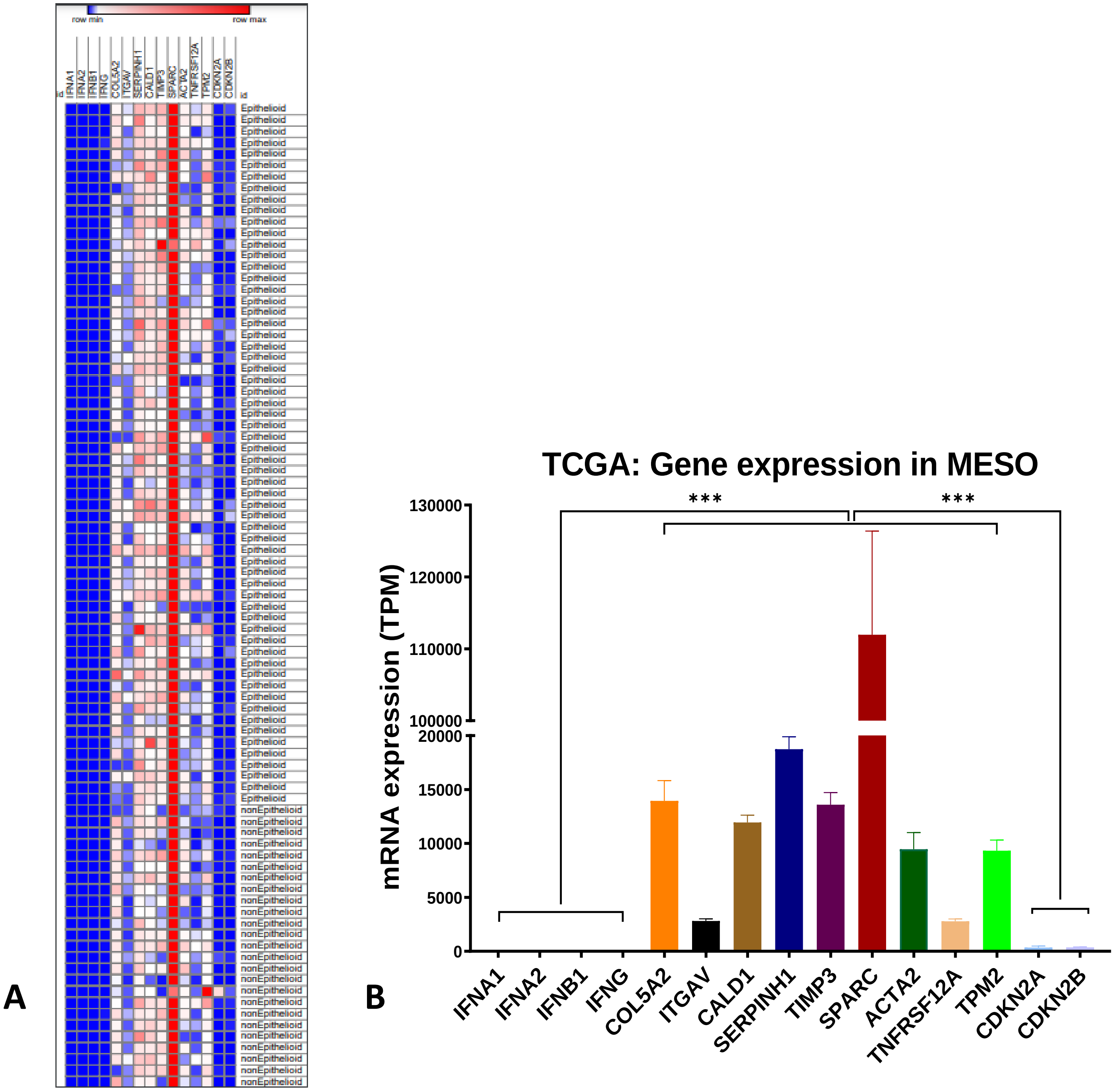

Supplement: Supplementary file 1 [file ijms-24-04264-s001.zip › ijms-2012297-supplementary.pdf]
